# Down Regulation of Genes Involved in T Cell Polarity and Motility during the Induction of Heart Allograft Tolerance by Allochimeric MHC I

**DOI:** 10.1371/journal.pone.0008020

**Published:** 2009-12-02

**Authors:** Wojciech Lisik, Neelam Tejpal, Yongquan Gong, T. Spencer Skelton, Malathesh Ganachari, Eric G. Bremer, Malgorzata Kloc, Rafik M. Ghobrial

**Affiliations:** 1 Department of General and Transplantation Surgery, Warsaw Medical University, Warsaw, Poland; 2 The Methodist Hospital and The Methodist Hospital Research Institute, Houston, Texas, United States of America; 3 Precision Biomarker Resources, Inc, Evanston, Illinois, United States of America; New York University School of Medicine, United States of America

## Abstract

**Background:**

The allochimeric MHC class I molecule [α1h1/u]-RT1.Aa that contains donor-type (Wistar Furth, WF; RT1u) epitopes displayed on recipient-type (ACI, RT1a) administered in conjunction with sub-therapeutic dose of cyclosporine (CsA) induces indefinite survival of heterotopic cardiac allografts in rat model. In vascularized transplantation models, the spleen contributes to graft rejection by generating alloantigen reactive T cells. The immune response in allograft rejection involves a cascade of molecular events leading to the formation of immunological synapses between T cells and the antigen-presenting cells.

**Methodology/Principal Findings:**

To elucidate the molecular pathways involved in the immunosuppressive function of allochimeric molecule we performed microarray and quantitative RTPCR analyses of gene expression profile of splenic T cells from untreated, CsA treated, and allochimeric molecule + subtherapeutic dose of CsA treated animals at day 1, 3 and 7 of post transplantation. Allochimeric molecule treatment caused down regulation of genes involved in actin filament polymerization (RhoA and Rac1), cell adhesion (Catna1, Vcam and CD9), vacuolar transport (RhoB, Cln8 and ATP6v1b2), and MAPK pathway (Spred1 and Dusp6) involved in tubulin cytoskeleton reorganization and interaction between actin and microtubule cytoskeleton. All these genes are involved in T cell polarity and motility, i.e., their ability to move, scan and to form functional immunological synapse with antigen presenting cells (APCs).

**Conclusions:**

These results indicate that the immunosuppressive function of allochimeric molecule may depend on the impairment of T cells' movement and scanning ability, and possibly also the formation of immunological synapse. We believe that these novel findings may have important clinical implications for organ transplantation.

## Introduction

Transplantation of genetically incongruous organ generates the immune response, which may eventually result in the destruction of the grafted tissue [1], [2]. Because currently used immunosuppressants induce impairment of the recipient's immune system, a major goal in transplantation is to prevent rejection by inducing tolerance while avoiding global immunosuppression [3], [4].

The administration of MHC class I allochimeric molecule [α1h1/u]-RT1.Aa that contains donor-type (Wistar Furth, WF; RT1u) immunogenic epitopes displayed on recipient-type (ACI, RT1a) sequences and produced by the alteration of the immunodominant determinant in the α1-helical region of class I MHC RT1.Aa to that of RT1.A1 and RT1.Au sequences induced indefinite survival of heterotopic cardiac allografts in rats when administered in conjunction with sub-therapeutic dose of cyclosporine (CsA) [5]–[8]. The results of several studies indicate that the soluble MHC class I proteins either directly inhibit T cell functions by receptor blockade [9] or induce apoptosis of activated CD8+ T cells [10]. Alterantively, the MHC molecules may modulate CD4+ T cells responses via phagocytosis and indirect presentation by antigen-presenting cells [11]. However, the cellular and molecular mechanisms underlying the immunosuppressive function of allochimeric [α1h1/u]-RT1.Aa molecule and the mechanism(s) by which this immunosuppressant regulates gene expressions of host's T cells and induce allograft tolerance remains largely unknown.

In vascularized transplantation models, the spleen contributes to graft rejection by generating alloantigen reactive T cells [12], [13]. It is well established that immune response relies on the ability of T cells to move, scan and to form the immunological synapse with the antigen presenting cells (APCs). Interaction of T cell with APC involves: active migration towards the APCs, the adhesive contact required to scan the surface of APC, and the polarization and redistribution of cytoskeleton which allows the close apposition of cell membranes necessary for T cell receptor (TCR) interaction with major histocompatibility complex [MHC; 14–25]. All these functions require polarized cytoskeleton and proper segregation of membrane, and adhesion and intracellular signaling proteins.

To find out which genes and molecular pathways are affected by the allochimeric molecule treatment, we have analyzed, using microarray and quantitative reverse transcription polymerase chain reaction (qRT PCR), the quantitative and temporal patterns of gene expression profile of splenic T cells in rats treated with allochimeric [α1h1/u]-RT1.Aa molecule in conjunction with sub-therapeutic dose of CsA in comparison to sub-therapeutic dose of CsA-treated and untreated rats at post-transplantation day 1, 3 and 7.

## Results

### Overview of Gene Expression Profiles

Gene expression profiles were generated from ACI host splenic T cells at 1, 3 or 7 days of post transplantation. Control transplanted animals received no treatment. Experimental transplanted animals were treated with sub-therapeutic dose CsA in conjunction with allochimeric peptide or with sub-therapeutic dose of CsA (see Material and Methods for details).

### Principal Components Analysis

**Figure 1:**
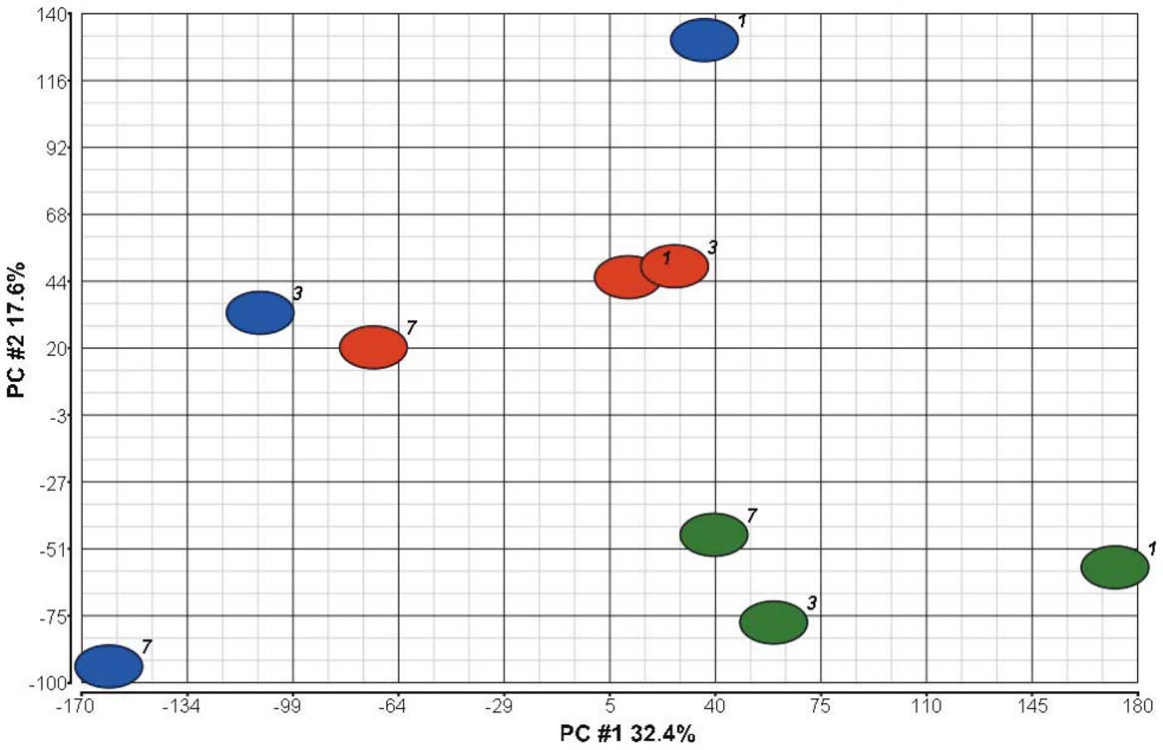
The principal component analysis (PCA). The allograft T Cell microarray data were analyzed by PCA using Partek Genomics Suite. 50% of the variation in samples was revealed in the first two principal components. The first principal component is represented by the X axis and the second principal component by the Y axis. Treatment type appeared to be major source of variation and is indicated by different colored ellipsoids. Green, untreated; Red, CsA only; and Blue, CsA plus peptide. The numbers next to each ellipsoid indicate the number of days post transplantation regardless of treatment type. The type of treatment appeared to be the most significant effect and the treatment time was another significant variable. Each treatment could be seen as distinct groups. Both the untreated (Green) and the cyclosporine treated (Red) samples seemed to form similar cluster shapes. Cyclosporine plus peptide (Blue) sample, on the other hand, was much more spread out. This could be due to another variable such as time after transplantation. Indeed, treatment time did appear to have some effect on the PCA since the ovoids representing 7 day-treatment samples were always to the left of the 1 or 3 days treatments.

Principal component analysis (PCA) is a standard tool used in modern data analysis, which simplifies (reduces) complex (multidimensional) and confusing data sets to lower dimensions (less complex). The goal of principal component analysis is to filter out the irrelevant “noise” in existing data set and to identify the most meaningful basis to present a data set. The principal components analysis (PCA) was performed using Partek Genomics Suite. PCA transforms the data to a new coordinate system such that the greatest variance by any projection of the data comes to lie on the first coordinate (called the first principal component), the second greatest variance on the second coordinate, and so on. Those characteristics of the data set that contribute most to its variance are retained. When the T Cell microarray data were analyzed by PCA, 50% of the variation in samples was revealed in the first two principal components. The type of treatment appeared to be the most significant effect and the treatment time was another significant variable (Fig. 1). The type of treatment appeared to be the most significant effect since each treatment could be seen as distinct groups. Both the untreated (Green) and the cyclosporine treated (Red) samples seemed to form similar cluster shapes. For example, the distance between each of the green ovoids is less than the distance between green (untreated) and red ovoids (cyclosporine treated). This suggests a greater variation due to treatment than time after treatment for these two conditions. Cyclosporine plus peptide (Blue) sample, on the other hand, was much more spread out. This suggests another variable such as time after transplantation has an important role in this treatment condition. Treatment time did appear to have some effect on the PCA for all treatment conditions since the ovoids representing 7 days treatment samples were always to the left of the 1 or 3 day treatments.

### Gene Changes Due to Treatment

Differential gene expression was determined in Partek Genomics Suite with the criteria described in Materials and Methods. Since post transplantation time also had an influence on the gene expression profiles, we examined the effect of treatment on gene expression for each day separately. Treatment with CsA and CsA + peptide were compared with the corresponding untreated control for that day (Table 1). Gene changes were divided into those that were unique to each treatment and those that were common to both the CsA and CsA + peptide treatments. Overall, more than 80% of these changes were genes down regulated compared to the untreated control groups, which is expected considering the immunosupressive function of CsA and CsA+peptide. The number of gene expression changes associated with sub-therapeutic doses of CsA was greatest at day 1 and quickly diminished by days 3 and 7. On the other hand, gene expression changes unique to CsA + peptide not only persisted but increased from 286 changes at day 1 to 683 changes at day 7 post treatment. Lists of individual gene changes for each day can be found in the supplementary tables.

**Table 1:** Number of differentially expressed genes at 1, 3 and 7 days post-transplantation from both CsA and CsA plus peptide treatments.

Treatment	Day 1	Day 3	Day 7
CsA only	490	78	105
CsA and Pep Overlap^1^	922	170	54
CsA and Pep only	286	584	683

1 Indicates the number of differentially expressed genes common to both CsA and CsA plus peptide treatment.

**Figure 2:**
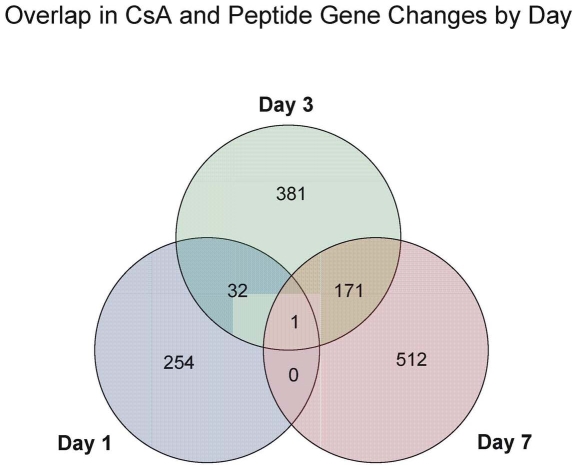
Overlap of CsA plus Peptide differential gene expression at 1, 3, and 7 days post-transplantation. The Venn diagram was created from the lists of differentially expressed genes unique to CsA plus peptide treatment (Table 1, row 3).

Since we were primarily interested in the genes involved in early events of immune response associated with the immunosuppressive function of allochimeric molecule, we focused our analysis on genes differentially expressed due to CsA + peptide treatment (Table 1, row 3). The increasing number of genes meeting the criteria for differential expression suggested a persistent immunsupression in the CsA + peptide treated animals. The increased number of changes also suggested that there might be early and additional late immunosuppressive effects of peptide treatment. The overlap of CsA + peptide gene changes within the given days can bee seen in Figure 2. There is surprisingly little overlap in these changes. Only 32 genes were differentially expressed at both 1 and 3 days post-treatment with CsA + peptide. When day 3 and 7 post- teatment with CsA+peptide were compared, the 171 genes common for both these days were differentially regulated. Only one gene was differentially regulated at all three time points. That gene was identified as an EST (AA800192) and was down regulated approximately 2 fold at all three days. This EST has sequence homologies with endogenous retroviral mRNA, LTR-repeat sequences and MHC class I in rat. Considering the fact that LTR repeats play a role not only in specific DNA rearrangement but also act as a MHC I gene promoter cis-acting activation response element [26]–[28] this finding is especially interesting.

### Early Gene Changes Associated with CsA and CsA + Peptide Treatments

The sub-therapeutic dose of CsA used in this study is not effective in preventing heart allograft rejection in rat model [5]–[8]. The same dose of CsA is, however, much more effective when administered in combination with the allochimeric molecule [5]–[8]. The numbers of gene changes shown in Table 1 is consistent with this idea. Initially (day 1), the CsA treatment downregulates a significant number of genes, which are probably the early responders, induced shortly after transplantation. Although this down regulation does not persist in CsA treatment, many gene changes do persist in the CsA + peptide treatment. Our overall assessment is that majority of the changes are not really persistent but rather can be divided into early and late changes. The lists of differentially expressed genes may provide some insight into the molecular mechanisms of action of these two immunosuppressants (Tables S1, S2, S3).

**Figure 3:**
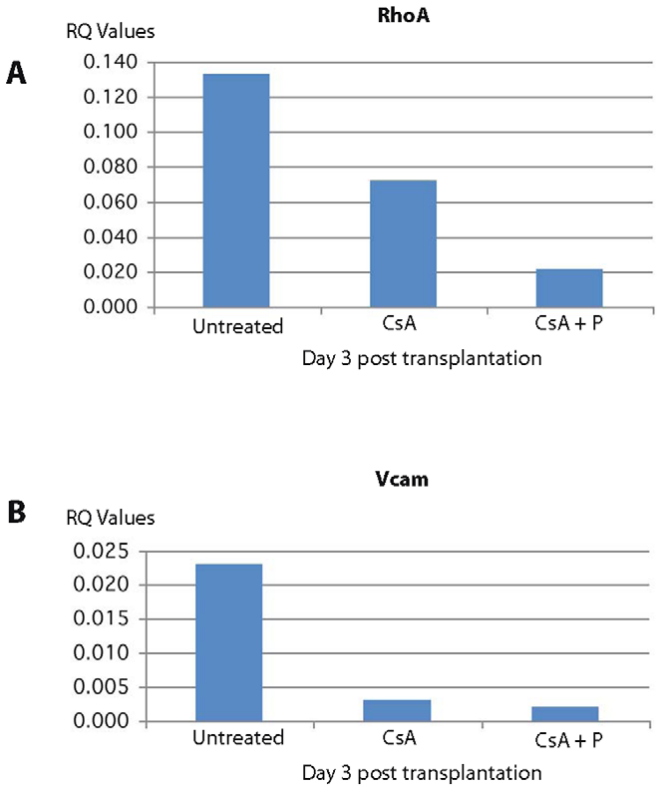
qRTPCR of expression of RhoA and Vcam in splenic T cells. The expression of RhoA (A) belonging to the family of Rho GTPases and inlvolved in the organization of cytoskeleton and T cell polarity, and Vcam (B) involved in cell adhesion is highly down regulated in rats treated with CsA plus allochimeric molecule (CsA+P).

**Figure 4:**
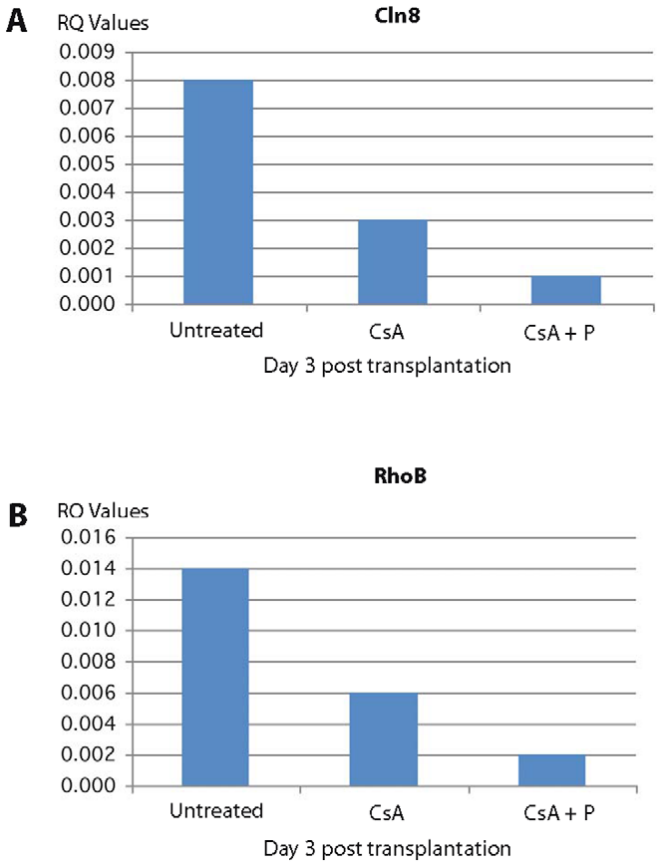
qRTPCR of expression of Cln8 and RhoB in splenic T cells. The expression of Cln8 (A) and RhoB (B) involved in vacuolar transport, vacuole organization and biogenesis is highly down regulated in rats treated with cyclosporine plus allochimeric molecule (CsA +P).

**Figure 5:**
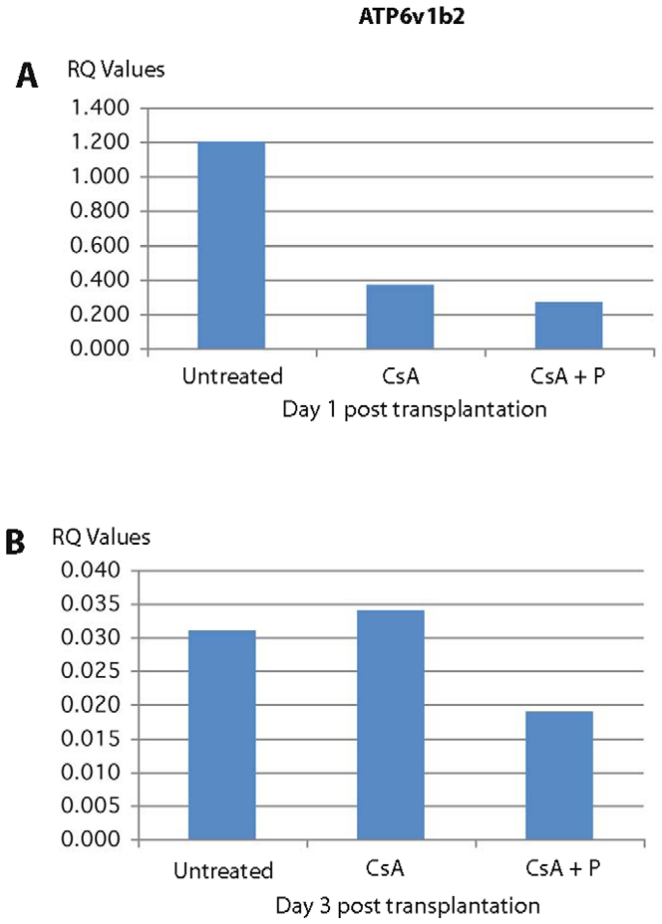
qRTPCR of expression of ATP6v1b2 in splenic T cells. The expression of ATP6v1b2 which is involved in vacuolar transport is highly down regulated in rats treated with cyclosporine plus allochimeric molecule (CsA +P).

**Figure 6:**
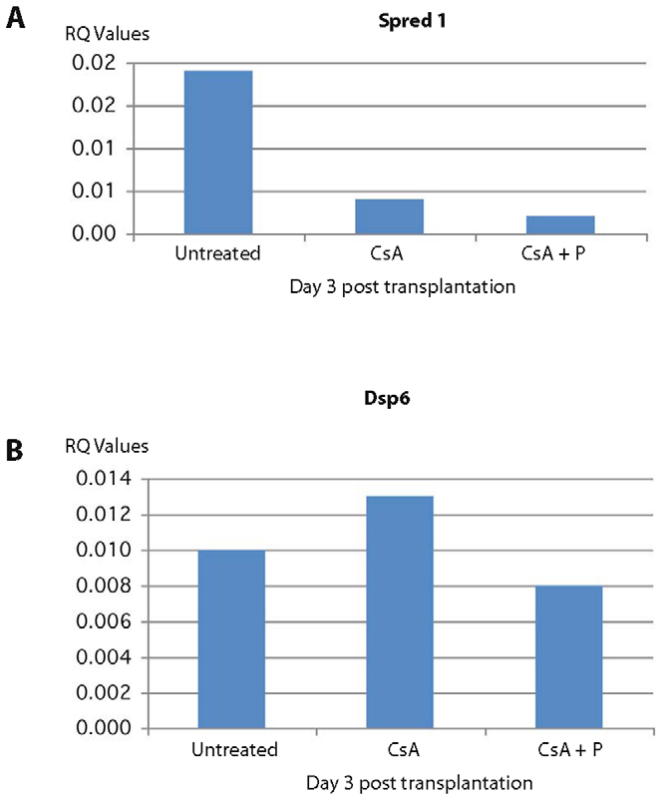
qRTPCR of expression of Spred 1 and Dsp6 in splenic T cells. The expression of Spred 1 (A) and Dsp6 (B), which belong to the MAPK pathway and regulate cellular proliferation and differentiation, is highly down regulated in rats treated with cyclosporine plus allochimeric molecule (CsA +P).

Most interestingly, among the genes down regulated at 1 day and 3 days post transplantation in CsA + peptide treatment were genes involved in actin filament polymerization and positive regulation of protein polymerization (RhoA and Rac1; 29–33), cell adhesion, and cell junction assembly and maintenance (Catna1, Vcam, and CD9; 17, 23, 24, 34–36), vacuolar transport, vacuole organization and biogenesis (Rhob, Cln8, ATP6v1b2; 37, 38–40) and MAPK pathway genes [Spred1 and Dusp6; 41, 42] involved in tubulin cytoskeleton reorganization and interaction between actin and microtubule cytoskeleton (Table 2). The down regulation of these genes was also confirmed independently by qRT PCR method (Figures 3, 4, 5, 6). All these genes are directly or indirectly involved in the pathways participating in T cell polarization, scanning ability and the formation of the immunological synapse (see Discussion).

**Table 2:** T cell polarity and motility gene changes.

Primary Gene Name	Affy Sequence ID	Gene Description	Accesion Number	Fold Change		
				Day 1	Day3	Day 7
*Actin Filament Polymerization*						
*positive regulation of protein polymerization*						
Rac1	1372513_at	gb:AI103616:Weakly similar to plysia ras-related homolog A2	AI103616	−1.532		
Rac1	1391048_at	Rattus norvegicus cDNA clone UI-R-BJ1-auh-f-06-0-UI 3, mRNA sequence	BE101148			−2.611
RhoA	1370130_at	ras homolog gene family, member A	NM_057132	−1.548		
*Cell Junction Assembly and Maintenance*						
*cell adhesion*						
Cd9	1371499_at	CD9 antigen	AI227627	−1.678		
Cd36	1367689_a_at	cd36 antigen	AF072411	−2.24	−1.978	
Cd36	1386901_at	cd36 antigen	NM_031561	−2.15	−2.369	
Catna1	1371921_at	Catenin (cadherin-associated protein) alpha 1	BM986272	−1.805		
Vcam1	1368474_at	vascular cell adhesion molecule 1	NM_012889	−1.727		
*Vacuolar Transport*						
*lysosomal transport*						
*vacuole organization and biogenesis*						
RhoB	1369958_at	ras homolog gene family, member B	NM_022542	−1.851		
Atp6v1b2	1387664_at	ATPase, H transporting lysosomal V1 subunit B2 sequence	NM_057213	−1.528		
Cln8	1389791_at	Ceroid lipofuscinosis, neuronal 8	AI179775	−1.718		
*MAPK Activity*						
Dusp6	1382778_at	gb:AI231350/UG = Rn.13146/UG_ TITLE = ESTs	AI231350	−1.563	−1.506	
Spred1	1377743_at	Rattus norvegicus cDNA clone UI-R-DO0-ciw-p-11-0-UI 3, mRNA sequence	BI292687	−1.791	−1.629	
Spred1	1374700_at	Rattus norvegicus cDNA clone UI-R-BS2-bdg-c-03-0-UI 3, mRNA sequence	BF388903	−1.578		
*Regulation of Natural Killer Cell Mediated Toxicity*						
Igsf4a	1379252_at	Rattus norvegicus cDNA clone UI-R-BO0-ahn-c-08-0-UI 3, mRNA sequence	AW522833	−1.551	−2.387	
Igsf4a	1376657_at	Immunoglobulin superfamily member 4A	BE117767		−3.343	

To identify additional functional related patterns from the differentially expressed gene lists, we performed a pathway analysis. This was done by comparison of our gene lists with canonical KEGG pathways using “Pathway Express” [43]. Pathway Express ranks the probability of a pathway being significantly affected with an impact factor [described in 43]. Based on an impact factor of three or greater we found 13 KEGG pathways to be affected from the CsA treated differential expressed gene list at day 1. In the CsA + peptide treatment at day 1 the additional 6 pathways were found to be affected (Table 3). These additional pathways also appeared to be consistent with the pathways involved in T cell polarization, migration and immunological synapse formation and functions.

**Table 3:** KEGG pathways overrepresented by differentially expressed genes at day 1 post-transplantation^1^.

Pathway Name	Impact Factor^2^	#Genes in Pathway	#Input Genes in Pathway	#Pathway Genes on Chip
Asthma	15.763	22	9	17
Hematopoietic cell lineage	14.948	76	17	68
Cell adhesion molecules (CAMs)	14.381	150	21	103
Graft-versus-host disease	12.854	59	10	28
Allograft rejection	12.135	61	10	30
Cytokine-cytokine receptor interaction	11.866	159	22	129
Authoimmune thyroid disease	9.259	69	9	33
Antigen processing and presentation	8.703	91	11	51
Type I diabetes mellitus	8.302	68	9	37
B cell receptor signaling pathway	7.221	63	10	51
Primary immunodeficiency	5.835	34	6	25
Natural killer cell mediated cytotoxicity	5.475	85	11	74
Complement and coagulation cascades	3.304	56	7	53
*Additional CsA+ Peptide Pathways*				
Toll-like receptor signaling pathway	5.266	90	10	71
Leukocyte transendothelial migration	4.692	115	12	101
T cell receptor signaling pathway	4.435	92	10	80
GnRH signaling pathway	3.414	86	9	81
MAPK signaling pathway	3.233	241	19	224
VEGF signaling pathway	3.099	66	7	60

1 Differential expression based on the criteria described in “Material and Methods” from both the CsA and CsA plus peptide treatment groups.

2 Impact factor calculated in Pathway Express [52].

3 Number of differentially expressed genes associated with the KEGG pathway.

### Comparison of 1 and 7 Day CsA + Peptide Treatments

Interestingly, pathway analysis revealed much more similarity between 1 day and 7 days after treatment with CsA + peptide. (Tables 3 and 4) Approximately one third of the significant pathways were identical. These included NK and B Cell activation pathways along with several of the associated signaling pathways, Toll like receptor, VEGF, and MAPK. In addition, pathways involved in cell adhesion remained significantly effected. Some of the new pathways appearing at day 7 were also related to Cell adhesion, Adherens junction, Focal adhesion and regulation of actin cytoskeleton. Majority of these pathways are involved in T cell polarity, migration and the formation of immunological synapse with antigen presenting cells.

**Table 4:** KEGG pathways overrepresented in CsA plus peptidetreatment at day 7 post-transplantation^1^.

	KEGG Pathway	Impact Factor^2^	# of Genes in Pathway	#Differentially Expressed Genes	# Genes on Chip
	Adherence junction	10.601	71	1	64
	VEGF signaling pathway	8.912	66	3	60
	Natural killer cell mediated cytotoxicity	6.32	85	2	74
	Focal adhesion	6.311	179	2	163
	B cell receptor signalling	5.602	63	2	51
	Axon guidance	5.18	113	2	93
	Pancreatic cancer	4.7	71	1	62
	Fc epsilon RI signaling pathway	4.642	70	1	68
**Day 7**	Wnt signaling pathway	4.601	124	2	112
	Reguation of actin cytoskeleton	4.135	194	1	170
	MAPK signaling pathway	4.096	241	1	224
	Renal cell carcinoma	3.746	68	1	62
	Biosynthesis of unsaturated fatty acids	3.695	23	1	20
	Toll-like receptor signaling pathway	3.622	90	1	71
	Colorectal cancer	3.61	79	1	72
	SNARE interactions in vesicular transport	3.564	36	1	31
	Cell adhesion molecules(CAMs)	3.284	150	1	103

1 Differential expression based on the criteria described in “Material and Methods” from only the CsA plus peptide treatment group.

2 Impact factor calculated in Pathway Express [52].

## Discussion

Our study of T cell expression profile showed that the expression levels of many genes either directly or indirectly involved in the molecular pathways responsible for T cell polarization, movement, scanning ability and the formation of the immunological synapse are down regulated during CsA + allochimeric molecule treatment when compared to CsA treatment alone or to the untreated control. The generation of immune response requires several highly specialized molecular and cellular events, which take place in sequential manner. First there is a migration of T cells toward the antigen presenting cells (APCs), followed by an initial adhesive contact between T cell and APC which allows for the scanning of APC surface by T cell, and finally the formation of the immunological synapse i. e. the close apposition of T cell and APC membranes necessary for the interaction between the T cell receptors (TCR) and major histocompatibility complex (MHC). The supramolecular spatial organization and highly dynamic clustering of the molecules in the immunological synapse allows T cell receptors to response to the antigen and to facilitate polarized secretion of cytotoxic granules and cytokines. All these events involve highly orchestrated polarization and rearrangements of cytoskeleton, membranes, vacuolar/endosomal compartments and cell adhesion and signaling molecules [14]–[21], [23], [25].

Our study showed that the following genes, directly or indirectly involved in all of the above events, are down regulated in T cells originating from allochimeric MHC molecule treated animals.

Rac1, RhoA and RhoB genes belong to the Rho family of GTPases. Rho GTPases cycle between an inactive, GDP-bound, and an active, GTP-bound state, and act like “molecular switches” controlling the dynamics of cell proliferation, movement, cytoskeleton and TCR signaling [29]–[33]. Engagement of the TCR has been shown to lead to the activation of Rac1 and Rac2 [44]. Consistent with a key role for Rac proteins in TCR mediated signaling, CD4+ T cells from Rac2−/− mice are defective in TCR mediated proliferation and IL-2 and IFN-γ production [45], [46]. TCR stimulation leads to the activation of RhoA, and blocking RhoA activation has been shown to decrease production of IL-2 [47]. In addition, it was shown that inhibition of ROCKs, key effectors of RhoA, caused a decrease in T cell proliferation and production of IL-2 and IFN-γ, and resulted in prolonged survival of cardiac allograft [48]. In the light of these data it is not surprising that immunosuppressive function of allochimeric molecule used in our study relies on the down regulation of genes belonging to Rho GTPase cascade.

The signaling Rac/Rho cascade triggered by TCR engagement is also tightly intertwined with the remodeling of cytoskeleton essential for decreased T cell rigidity that enables closer contacts between the T cell and the APC and the formation of immunological synapse [49], [50]. Rac and Rho are also involved in the integrin-mediated adhesion between T cells and APCs [49], control of actin polymerization at the T cell/ APC interface, and in proper clustering of lipid rafts and TCR at the immunological synapse [50], [51], [52]. In addition, Rho GTPases are involved in the acquisition of a polarized morphology of T cells i.e. in the processes that are critical for the movement and migration of T cells [49], [53], [54]. Unlike Rac and RhoA, the RhoB is an immediate early response gene, which has a short half-life [37], is associated with the plasma membrane and endosomal compartments, and similar to Cln8 (Ceroid-lipofuscinosis, neuronal 8) and ATP6v1b2 (ATPase, hydrogen-transporting, lysosomal, V1 subunit B, isoform 2) genes, it is involved in the trafficking of receptors and signaling molecules through the vesicular/endocytic pathway [39], [40].

Considering all of these data we believe that the immunosuppressive function of allochimeric molecule may depend on the impairment of T cell motility and scanning ability, T cells adhesion with APCs, and the proper TCR clustering, and possibly also the formation of immunological synapse.

Another genes down regulated in allochimeric molecule treatment are Catna1, Vcam and CD9, which are involved in intercellular adhesion, interaction with cytoskeleton, signaling and regulation of inflammatory response. Vcam-1 (vascular cell adhesion molecule-1 also known as CD106) is a membrane protein and an integrin ligand, which mediates leukocyte-endothelial cell adhesion and signal transduction, and is an important factor in the initiation of an inflammatory response [35], [55]. Indeed, conditional Vcam-1 mutant mice have an impaired immune response [56], and the over expression of Vcam-1 is associated with several chronic inflammatory diseases [57], [58]. In addition, VCAM-1 is linked to actin cytoskeleton in immunological synapse [17], [19], [25]. Catna1 (catenin alfa 1; cadherin-associated protein; Ctnna1) is an intracellular protein that associates with cadherins and cytoskeleton and is required for the formation and maintenance of functional intercellular adhesion complex [34]. CD9 is a member of the tetraspanin superfamily (which also includes MHC molecules). Tetraspanins are known to be involved in cell adhesion, motility, and cell differentiation and activation. CD9, which is expressed on most mature and naïve T cells, associates with various cell surface molecules including co-stimulatory CD5 molecule [36]. We suggest that the down regulation of these three genes in allochimeric treated animals maybe responsible for the impairment in T cell/APC adhesion, T cell motility and immune response.

Allochimeric molecule treatment also causes a down regulation of Mitogen-activated protein kinases (MAPK) pathway genes Dusp6 and Spred1. The Dusp6 (Dual specificity phosphatase 6) is a phosphatase, which inactivates members of the mitogen-activated protein (MAP) kinase superfamily (MAPK/ERK, SAPK/JNK), which are associated with cellular proliferation and differentiation. Recent studies on T cells indicate that Dusp5 and DuspP6 are induced by IL-2 [42]. A novel gene Spred-1 (Sproutyrelated Ena/VASP homology 1-domain-containing protein-1) is involved in the inhibition of the Ras/Raf-1/ERK pathway impairing the growth-factor-mediated activation of ERK1/2 kinases [41]. Studies of Spred1 expression in T cells showed that Spred-1 is highly up regulated in the tumor-infiltrating CD8^+^ T cells and that TGF-β modulates the infiltrating function of CD8^+^ T cells via TCR signaling and Spred1 expression [41].

In conclusion, the T cells gene expression changes described in our studies indicate multiple protective influences of allochimeric peptide and will provide basis for further studies aimed at understanding of pathways and mechanisms involved in the immunosuppression and heart graft survival.

## Materials and Methods

### Animals

Adult male inbred Wistar Furth (WF; RT1.Au) and ACI (RT1.Aa) rats (180–250 gm) were purchased from Harlan Sprague Dawely (Indianapolis, IN). Heterotopic cardiac transplants were placed intra-abdominally as described previously [5]. There were three experimental groups: 1. Transplantation control group without any treatment, 2. Transplantation in the presence of sub-therapeutic dose of CsA (acute rejection) which consisted of 3 day course of oral cyclosporine delivered by gavage feed (CsA, 10 mg/kg/day; day 0–2). Transplantation in the presence of sub-therapeutic dose of CsA supplemented with allochimeric molecule (CsA + peptide). Allochimeric peptide [α1h1/u]-RT1.Aa (GenWay, San Diego CA; 1 mg/kg) was delivered through the portal vein into ACI recipients of WF hearts at the time of transplantation in addition to a 3 days course of oral cyclosporine delivered by gavage feed (CsA, 10 mg/kg/day; day 0–2). All experiments were performed according to the The Methodist Hospital Research Institute animal care and use NIH standards as set forth in the “Guide for the Care and Use of Laboratory Animals” (DHHS publication No. (NIH) 85–23 Revised 1985). The Institution also accepts as mandatory the PHS “Policy on Humane Care and Use of Laboratory Animals” and NIH “Principles for the Utilization and Care of Vertebrate Animals Used in Testing, Research and Training.

### T cells Isolation and FACS Analysis

Spleens from ACI host rats were harvested at 1, 3 and 7 days post-transplantation. Cell suspension was made by passing spleen through a cell strainer using 3cc syringe. Cells were treated with lysing reagent (Becton Dickenson ) to remove the red cells and then washed twice with complete media (10%Fcs/1640RPMI). T cell population was purified via a positive T cell isolation kit using magnetic anti-T cell micro beads (Miltenyl Biotech) and purity of T cells was confirmed by FACS analysis i.e. T cells were stained with BD Pharmingen (Franklin Lakes, NJ) reagents including FITC-conjugated mouse anti- rat CD3 and CD4 antibodies for 15 minutes at the room temperature and then washed three times with PBS and analyzed using FACScan flow cytometer (Becton Dickenson, San Jose, CA).

### RNA Isolation

For RNA isolation isolated T cells were pelleted, immediately placed in RNA Later (Applied Biosystems/Ambion, Austin, TX) and after overnight infiltration at 4°C they were kept in RNA Later at −70°C until the isolation of total RNA. T cell pellets were homogenized using a TissueLyser (Qiagen, Valencia, CA) and RNA was isolated using the RNeasy Mini Kit (Qiagen, Valencia, CA) according to the manufacturer's recommendations. The quantity, purity and integrity of RNA were evaluated using a NanoDrop ND-1000 spectrophotometer (Nanodrop Technologies, Wilmington, DE) and an Agilent Bioanalyzer (Agilent Technologies, Palo Alto, CA).

### Quantitative RT PCR

Total RNA was isolated from purified spleenic T cells using RiboPure kit (Applied Biosciences, Foster City, CA). Complementary DNA (cDNA) was made using High Capacity cDNA Reverse Transcription Kit (Applied Biosciences, Foster City, CA). The cDNA was used to determine the expression levels of house keeping actin β (ACTβ) gene and gene of interest. Total RNA (1000 ng) was reverse transcribed (RT) and PCR amplified using the High Capacity cDNA Reverse Transcription Kits (Applied Biosystems) according to the manufacturer's protocol. The RT reaction consisted of a 10 min incubation at 25°C, 120 min incubation at 37°C, followed by a 5 min 85°C termination step, and the resulting complementary DNA (cDNA) was stored at − 20°C. For Real time PCR amplification samples were run in duplicate and only 1 gene was analysed per reaction. The cDNA template reaction contained Assay On Demand Gene Expression primers (see below; Applied Biosystems, Foster City, CA) and TaqMan® Fast Universal PCR no AmpErase UNG master mix (Applied Biosystems, Foster City, CA). Reactions were heated to 95°C for 10 min, followed by 40 amplification cycles of 95°C for 15 s and 60°C for 1 min using Applied Biosystems 7500 Standard System. The amount of target mRNA relative to housekeping gene mRNA was expressed as fold incerase /decrease. Relative changes were measured using real time PCR in the 7500 Fast or Standard Real Time PCR System (version 1.3.1). To calculate the relative quantity (RQ) of particular gene, 2-delta delta ct method implemented in the software was used. The data are presented as the fold change (RQ values) in gene expression level normalized to endogenous reference gene.

### PCR Primers

All PCR primers were purchased from SABiosciences (Frederick, MD, USA): Spred 1, cat #PPR55168; Vcam, cat#PPR45334; RhoA, cat# PPR56555A; RhoB, cat# PPR42656A; Rac, cat #PPR48291A; CD9, cat# PPR42666A; Catna, cat# PPR48584A; Cln8, cat# PPR42624A; ATP6v1b2, cat# PPR44008A; Dusp6, cat# PPR43415.

### Microarray

The microarray hybridization and analysis were performed by Cogenics (Morrisville, NC) according to the manufacturer's protocol (Affymetrix, Santa Clara, CA). Total RNA from each sample was converted to double-stranded cDNA with the BioarrayTM Single-Round RNA Amplification and Labeling Kit (Enzo Life Sciences), Double-stranded DNA purified with Purification Kit (Enzo Life Sciences was ttranscribed in vitro using the BioarrayTM HighYieldTM RNA Transcript Labeling Kit (Enzo Life Sciences). For each sample, biotinylated cRNA spiked with bioB, bioC, bioD and cre (Hybridization Control) was hybridized to an Affymetrix Rat 230 2.0 Microarray for 16 hours at 45°C. After hybridization, washing and staining, arrays were scanned with an Affymetrix GeneChip® Scanner 3000. Quality checks and data analyses were performed using Affymetrix GeneChip Operating Software (GCOS) and Expression Console. All microarray data reported in the manuscript are described in accordance with MIAME guidelines.

### Microarray Data Analysis

The processed image file of the Affymetrix Rat 230 2.0 array contains over 31,000 probe sets representing approximately 28,700 well-substantiated rat genes (Affymetrix; www.affymetrix.com). The probe set level data were analyzed with either Rosetta Resolver Gene Expression Data Analysis System (Rosetta, Seattle, WA) or Partek Genomics Suite, version 6.4, build 6.09.0129 (Partek, St. Louis, MO). The criteria for identification of differentially expressed transcripts was a log ratio p-value<0.001, an absolute fold change greater than 1.5, and a log (10) intensity measurement >−1.6. Enrichment of biological pathways for differentially expressed genes was determined using Pathway Express [43]. The microarray data used in this study were deposited in the National Center for Biotechnology Information (NCBI) Gene Expression Omnibus (GEO) with the accession number GSE15074 (www.ncbi.nlm.nih.gov/projects/geo).

## Supporting Information

Table S1 Day 1 gene changes.
(0.79 MB XLS)

Table S2 Day 3 gene changes.
(0.41 MB XLS)

Table S3 Day 7 gene changes.
(0.43 MB XLS)
